# A Rare Case Presentation of Apixaban-induced Nontraumatic Spinal Subdural Hematoma

**DOI:** 10.7759/cureus.6743

**Published:** 2020-01-22

**Authors:** Saif Faiek, Noor Idilbi Wazzan, Sreeja Sompalli, Aditya Bansal, Partha Hota

**Affiliations:** 1 Internal Medicine, AtlantiCare Regional Medical Center, Atlantic City, USA; 2 Internal Medicine, Trinitas Regional Medical Center, Elizabeth, USA; 3 Critical Care Medicine, AtlantiCare Regional Medical Center, Atlantic City, USA; 4 Radiology, Atlantic Medical Imaging, Galloway, USA

**Keywords:** apixaban, spinal subdural hematoma, atrial fibrillation, anticoagulants

## Abstract

A spinal subdural hematoma is a rare clinical entity and an uncommon urgent complication that can be associated with the use of vitamin K and less commonly nonvitamin K oral anticoagulants. It is considered a neurological emergency requiring prompt diagnosis and surgical intervention in the majority of the cases. Herein, we present an 84-year-old male patient with a history of nonvalvular atrial fibrillation on apixaban who presented with complaints of bilateral lower extremity weakness, severe back pain, and urinary retention. His lumbar and thoracic spine images showed a diffuse spinal subdural hematoma. Urgent neurosurgical intervention was performed with minimal improvement in his symptoms postoperatively. We report a case of spontaneous spinal subdural hematoma related to apixaban use with relevant literature review.

## Introduction

Spinal subdural hematoma (SSDH) is a very rare neurological emergency that accounts for 4.1% of all intraspinal hematomas [1]. The exact etiology is still controversial with the hypothesized mechanism of a sudden increase of pressure in thoracic and/or abdominal cavities raising the pressure inside the subdural vessels with a subsequent rupture and formation of a hematoma [2]. Risk factors include arteriovenous malformations, coagulopathy, therapeutic anticoagulation, underlying neoplasms, or following a spinal puncture. Nonvitamin K oral anticoagulants (NOACs) have been reported on multiple case reports as a risk factor for SSDH [3]. 

## Case presentation

**Figure 1:**
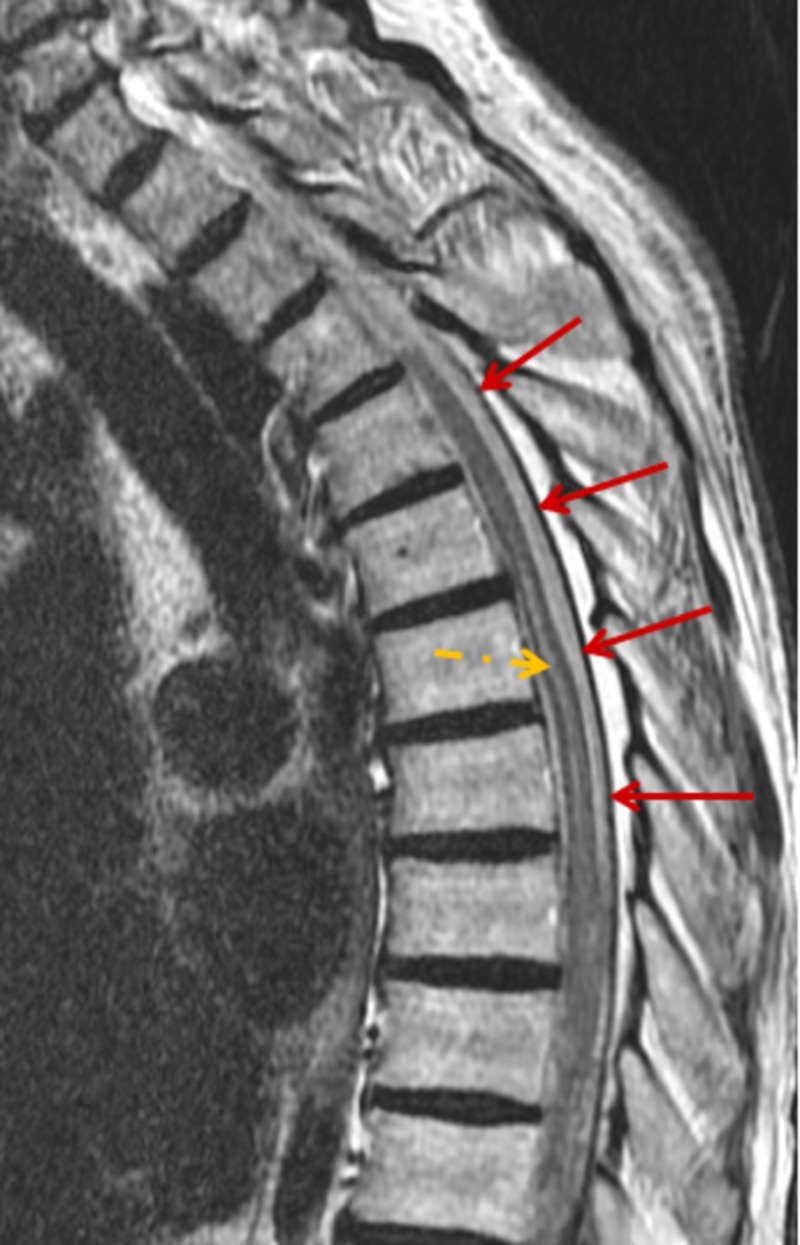
MRI scan, T2-weighted sagittal view demonstrates diffuse subdural intraspinal hematoma throughout the thoracic spine (red arrows) with spinal cord edema (yellow arrow).

**Figure 2:**
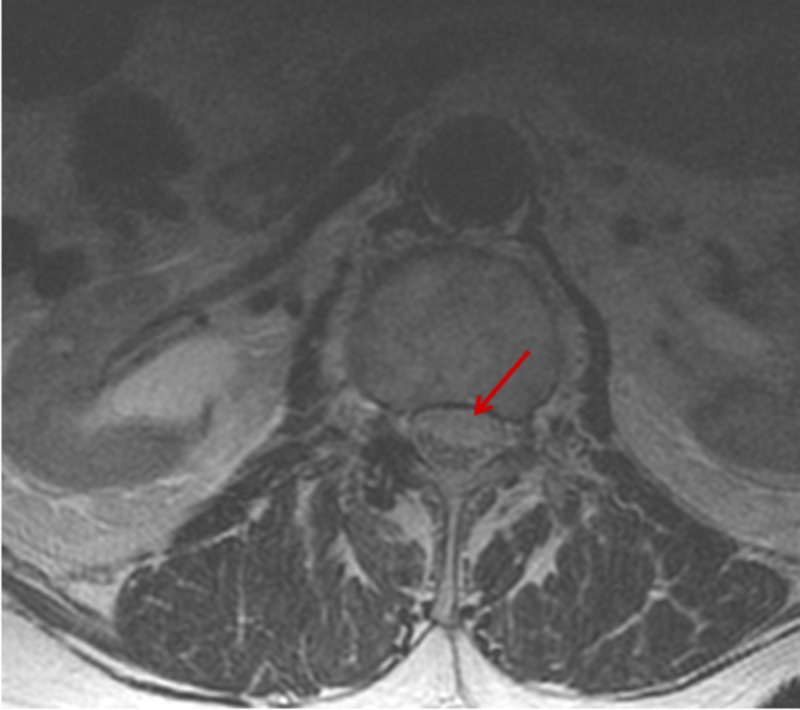
MRI scan, T2-weighted axial view of the lumbar spine demonstrates subdural intraspinal subdural hematoma with mild ventral mass effect on the cauda equina nerve roots (red arrow)

**Figure 3:**
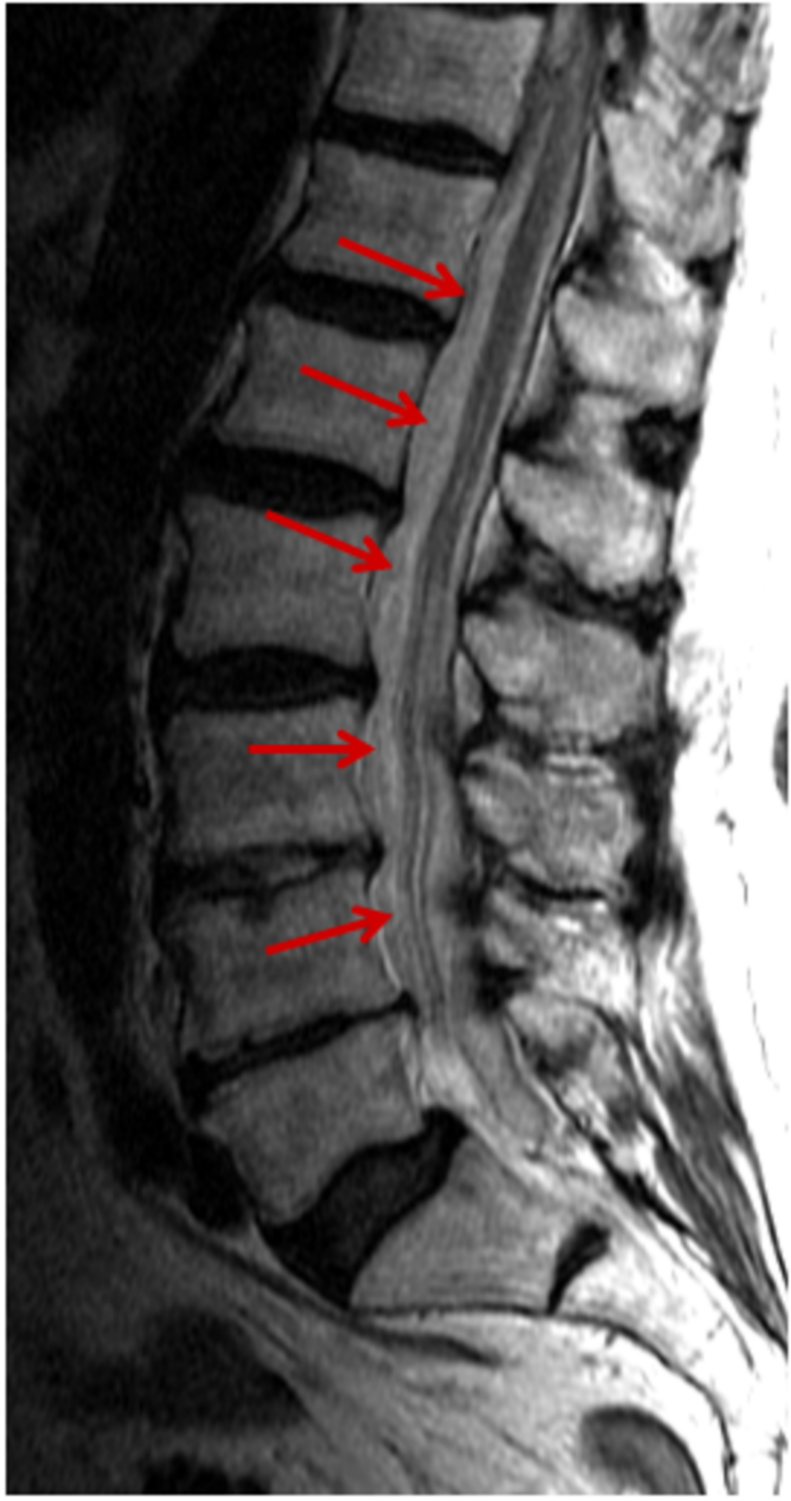
MRI scan, T2-weighted sagittal view demonstrates diffuse subdural intraspinal hematoma throughout the lumbar spine (solid arrows).

An 84-year old Caucasian male patient presented to the emergency department with bilateral lower extremity weakness, severe back pain, and urinary retention, which have started just prior to the presentation. He has a history of chronic nonvalvular atrial fibrillation with a CHA2DS2-VASc score of 4 on 5 mg of apixaban twice a day. His other past medical history is significant for coronary artery disease status post-four-vessel coronary artery bypass graft, hypertension, dyslipidemia, and ongoing tobacco use. The initial physical exam revealed a loss of pain, pressure, sensation, and motor function below T8 level. MRI of the brain and CT angiogram of chest and abdomen were normal. MRI spine revealed diffuse subdural hematoma extending from the superior endplate of the T12 to the cauda equina compressing the cauda equina and displacing the conus medullaris with extension to the cervical-thoracic junction (Figures 1-3). 

Immediate neurosurgical consultation determined the necessity of emergent surgical thoracolumbar laminectomy with durotomy and nerve decompression, while the reversal agent andexanet alfa was administered preoperatively. Neurophysiological monitoring revealed no signals or electrical activity in the lower extremities. Laminectomy of the T8-L3 thecal sac exposed significant blood clotting with wound irrigation, superior and inferior, and the cauda equina nerves were manipulated due to thick loculated clots. Anticoagulation was held, and the patient was transferred postoperatively into the ICU for further evaluation and monitoring. Neurological symptoms minimally improved in the following days, and the patient was discharged to a rehabilitation center for further treatment. On follow up, four months after the procedure, he continues to do physical therapy and still has residual paraplegia.

## Discussion

NOACs are increasingly used as alternatives to warfarin, as the latter has many potential limitations and side effects, including food drug interactions, delayed onset of action, narrow therapeutic window, and the need to close monitoring of the international normalized ratio plus a number of associated hemorrhagic adverse events. Apixaban is a direct factor Xa inhibitor and one of the new oral anticoagulants that has been shown to be effective in the reduction of stroke and embolic events in patients with nonvalvular atrial fibrillation [2]. A significant adverse effect of the drug is intracranial hemorrhage and traumatic spinal hematoma, although less common when compared with warfarin, especially in patients undergoing neuroanesthesia or spinal puncture [4].

The causes for spinal hematoma can be divided into post-traumatic, iatrogenic (following surgery or lumbar puncture), and rarely spontaneous, especially in cases with underlying malformations (e.g., vascular malformation and neoplasm) and coagulation disorders. There have been limited reported cases of spontaneous SSDH associated with NOACs use, such as apixaban [5,6]. The most common sites of SSDH have been published in the thoracic spine (40%), followed by the cervicothoracic and thoracic lumbar spine [6].

The most common clinical manifestation is acute severe back pain with radicular signs, which is frequently accompanied by sensory, motor, and autonomic dysfunction, including erectile dysfunction and urinary retention [7]. Domenicucci et al. reported the most common symptoms to be motor deficits (57% of patients), spinal pain (45% of patients), radicular pain (22% of patients), and paresthesia [8]. Since it can be life-threatening, it is essential that physicians consider spinal hematoma in a patient on NOACs presenting with acute neurological symptoms. MRI still considered the modality of choice in evaluation for SSDH due to its capability of visualizing spinal hematoma as well as spinal cord anatomy and pathologies [7]. Due to the rarity of this condition, the definite management has not been established yet, and it usually includes emergent surgical evacuation with decompressive laminectomy and discontinuation of NOACs. Better outcomes can be expected with early neurological evaluation and intervention [8]. Conservative management might be considered in cases with mild neurological deficits, rapid spontaneous recovery, or high-risk surgical candidates [1,2,7].

## Conclusions

With the increasing use of NOACs, especially apixaban, physicians should be aware of the increasing incidence of spontaneous nontraumatic spinal hematoma in patients on anticoagulants. It is an urgent complication that can be life-threatening or can lead to permanent disability. The prognosis depends on the rapid diagnosis using MRI and surgical intervention in severe cases. Since there is limited literature documenting this complication of NOACs, treatment protocols for such cases would be extremely beneficial.
